# Multimodal Imaging Features of Schnyder Corneal Dystrophy

**DOI:** 10.1155/2020/6701816

**Published:** 2020-03-23

**Authors:** Wassim Ghazal, Cristina Georgeon, Kate Grieve, Nacim Bouheraoua, Vincent Borderie

**Affiliations:** ^1^Centre Hospitalier National d'Ophtalmologie des 15-20, Paris, France; ^2^Sorbonne Université, GRC n°32, Transplantation et Thérapies Innovantes de la Cornée, CHNO des 15-20, Paris, France; ^3^CIC 1423, INSERM, Sorbonne Université, CHNO des 15-20, Paris, France

## Abstract

**Objective:**

To describe the multimodal imaging of Schnyder corneal dystrophy.

**Methods:**

Seven eyes of seven patients (5 female and 2 male patients) aged 52 to 92 years were included in this prospective observational study. Diagnosis of SCD was confirmed by histology after keratoplasty. In vivo multimodal imaging consisted of spectral domain-optical coherence tomography with cross sections, en face scans, corneal pachymetry, and epithelial mapping, and in vivo confocal microscopy was recorded. Ex vivo full-field optical coherence tomography scans of two corneal buttons were analyzed. The seven corneal buttons obtained during penetrating or deep anterior lamellar keratoplasty were processed for light microscopy.

**Results:**

Slit-lamp examination showed central stromal opacities, arcus lipoides, and midperipheral haze. Corneal crystals were found in 2 out of 7 eyes. SD-OCT cross sections and en face scans showed diffuse hyperreflectivity of the anterior, mid, and posterior stroma with a maximum in the anterior stroma, hyporeflective stromal striae, and epithelial hyperreflectivity. Central corneal thickness ranged from 507 to 635 *μ*m. IVCM revealed hyperreflective deposits in the epithelium and throughout the stroma, thin subepithelial nerves, and needle-shaped and rectangular crystals. Keratocyte nuclei were rare or undetectable. FF-OCT scans confirmed the presence of small round and needle-shaped hyperreflective deposits in the epithelium and stroma. Histology revealed vacuolization of the basal epithelial cells and empty interlamellar stromal vacuoles.

**Conclusion:**

High-resolution multimodal imaging demonstrates the characteristic features of SCD which involve both the corneal epithelium and stroma, and it provides diagnosis confirmation even in eyes with no visible corneal crystals at slit-lamp examination.

## 1. Introduction

Schnyder corneal dystrophy (SCD) is a rare autosomal dominant disease that results in deposits of cholesterol and phospholipids in the corneal stroma [1–3]. Mutations in the UBIAD1 gene (genetic locus 1p36) were shown to be responsible for the disease [4, 5]. Association with metabolic disorders, such as hypercholesterolemia, has been suggested [6]. The main clinical features of the dystrophy include corneal crystals, central discoid opacities, arcus lipoides, and stromal haze. However, crystals are only found in 54% of cases [7]. SCD is a bilateral slowly progressive disease, and corneal features are age-related. Central opacities and/or crystals are often observed in patients aged 23 years or younger, arcus lipoides appears in patients aged between 23 and 38 years, and finally midperipheral stromal haze can develop in patients after the age of 38, causing cloudiness of the cornea [8]. Visual acuity and photopic vision decrease with age.

The diagnosis of SCD is established with slit-lamp examination and confirmed by histopathology and genetic testing. Light microscopy analysis reveals abnormal deposits of both intracellular and extracellular esterified and unesterified phospholipids and cholesterol in basal epithelial cells, Bowman layer, and stroma [9]. Lipid accumulation has also been observed with transmission electron microscopy [10].

The absence of visible corneal crystals makes the diagnosis more challenging. Modern imaging techniques such as spectral domain-optical coherence tomography (SD-OCT) and in vivo confocal microscopy (IVCM) allow a more precise approach to the disease at all stages, especially when crystals are absent. Both imaging techniques have become the modality of choice for in vivo assessment of corneal disorders.

Full-field optical coherence tomography (FF-OCT) is a noninvasive technique that provides detailed ex vivo en face and cross-sectional imaging of corneal structures with a theoretical resolution close to 1 *μ*m in all three dimensions. It allows a cellular resolution very similar to histology [11, 12].

The objective of the present study was to report the distinguishing features of SCD in seven patients using SD-OCT, IVCM, and FF-OCT and the histological findings in corneal buttons obtained after keratoplasty.

## 2. Materials and Methods

This was a prospective observational study which was part of the MicroEye project. Informed consent was obtained, and Institutional Review Board (IRB) approval was obtained from the Patient Protection Committee, Ile-de-France V (14944). The study was carried out according to the tenets of the Declaration of Helsinki and followed international ethical requirements for human tissues.

Inclusion criteria were as follows: corneal disorder requiring transplantation, preoperative assessment with multimodal imaging (i.e., SD-OCT and IVCM), postoperative assessment of the recipient corneal button with histology and FF-OCT, diagnosis of Schnyder corneal dystrophy confirmed by histological analysis of the corneal button, and written informed consent. Exclusion criterion was as follows: nonconclusive histological analysis of the corneal button. Recipient age and sex were not included in inclusion/exclusion criteria.

We included 7 eyes of 7 patients who had been previously clinically diagnosed with SCD between 2013 and 2017 and who were referred for transplantation in our institution. The diagnosis was confirmed by histology of the corneal button after keratoplasty in all eyes. Best-corrected visual acuity (BCVA), slit-lamp photographs, and central corneal thickness were recorded. All patients underwent SD-OCT with epithelial mapping, as well as IVCM imaging for six patients before keratoplasty. Corneal buttons obtained from either penetrating or deep anterior lamellar keratoplasty were processed for light microscopy analysis. FF-OCT was performed before processing of tissue for histology on 2 out of 7 corneal buttons.

SD-OCT cross-sectional and en face scans were obtained using the RTVue® device (Optovue, Inc., Fremont, CA), with an axial theoretical resolution of 5 *μ*m. In vivo corneal morphology was assessed using the Heidelberg Retina Tomograph II with a Rostock Cornea Module (HRT II RCM) (Heidelberg Engineering GmbH, Dossenheim, Germany). 400 × 400 *μ*m en face images of all the corneal layers were acquired. The LightCT FF-OCT device (LLTech, Paris, France) was used to analyze corneal buttons. Stacks of 800 *μ*m × 800 *μ*m en face images were captured in the central zone of the cornea, and cross-sectional views were reconstructed from the 3D data stack. All corneal buttons were fixed in formaldehyde (10%) and embedded in paraffin. Multiple sections were then stained with hematoxylin-eosin-safran (HES), periodic acid-Schiff (PAS), and Masson's trichrome stain and observed with a light microscope.

A retrospective healthy group was used as control. It included five eyes of patients with normal corneas imaged with FF-OCT and IVCM and two normal human donor corneas imaged with FF-OCT.

## 3. Results

**Figure 1:**
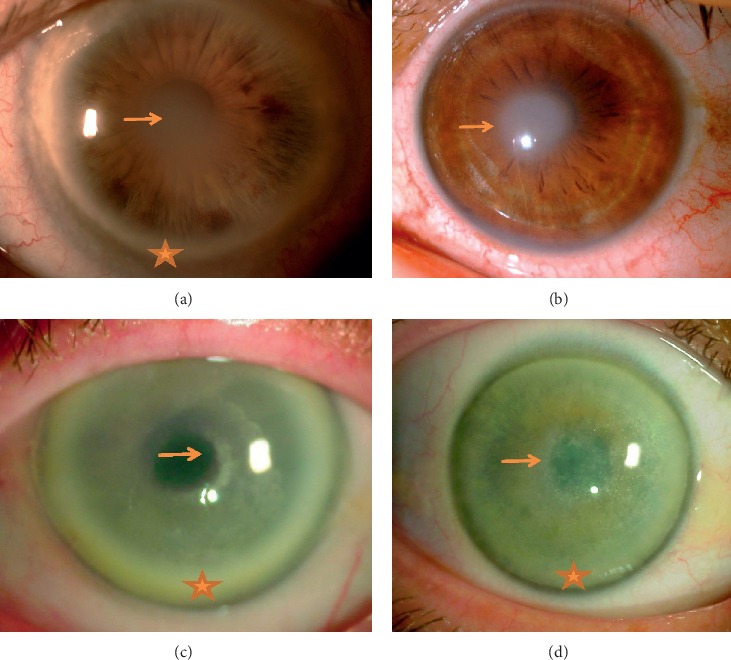
Slit-lamp photographs of Schnyder corneal dystrophy patients showing central opacities (arrows), midperipheral stromal haze, and peripheral arcus lipoides (stars) in most patients. Corneal crystals were less frequently observed. (a) Patient #1, central stromal opacity with arcus lipoides. (b) Patient #4, arcus lipoides was absent in the youngest patient. (c) Patient #6, central opacities with diffuse crystals and midperipheral stromal haze. (d) Patient #7, prominent central crystals and stromal haze.

Seven eyes of five female and two male patients aged 52 to 92 years were included in our study. Preoperative best-corrected visual acuity ranged from 20/2000 to 20/50. All patients had a family history of cloudy corneas. Slit-lamp examination revealed central opacities, midperipheral stromal haze, and peripheral arcus lipoides (Figure 1(a)) in all seven patients, except in the youngest patient who only presented with central disciform opacity (Figure 1(b)). Corneal crystals were observed in only 2 out of 7 patients (Figures 1(c) and 1(d)). The mean (±standard deviation) central corneal thickness was 563 ± 44 *μ*m (range, 507–635 *μ*m). The clinical data, corneal pachymetry, and epithelial mapping of all patients are shown in Table 1.

**Figure 2:**
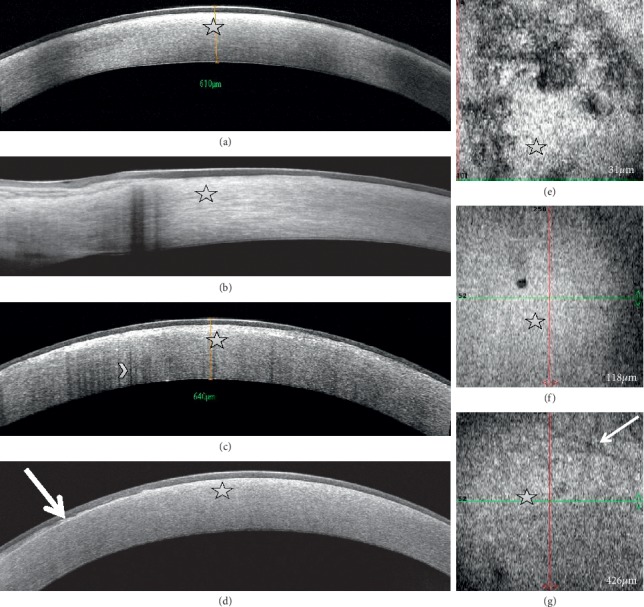
Cross-sectional and en face spectral domain-optical coherence tomography scans showing diffuse hyperreflective corneal stromal opacities (arrows), maximum in the anterior stroma and expanding to the limbus in eyes with Schnyder dystrophy. (a) Central diffuse stromal hyperreflectivity. (b) Limbal and peripheral corneal stromal hyperreflectivity. (c) Diffuse optical shadow from the stromal side (arrowhead; patient #7). (d) Thick hyperreflective corneal epithelial basement membrane insinuating into the epithelium (thick arrow) (patient #5). En face scans reveal epithelial (e), anterior stromal (f), and posterior stromal (g) hyperreflectivity. Stromal striae are visible in the posterior stroma (thin arrow).

Cross-sectional SD-OCT showed diffuse hyperreflective corneal stromal opacities which were maximum in the anterior stroma (Figure 2(a)), expanding to the limbus when arcus lipoides was present (Figure 2(b)). In one patient with prominent corneal crystals, diffuse posterior optical shadow from the stromal side was observed (Figure 2(c)). Epithelial mapping revealed areas of thick and irregular epithelium, ranging from 62 to 76 *μ*m within the central 5 mm zone. In one case, SD-OCT showed a thick hyperreflective epithelial basement membrane (Figure 2(d)) that appeared to expand into the epithelium in en face OCT scans. En face OCT scans revealed diffuse hyperreflective opacities located in the epithelium (Figure 2(e)), anterior (Figure 2(f)), and mid and posterior stroma (Figure 2(g)) in all patients, and hyporeflective stromal striae in the posterior stroma in three patients (Figure 2(g)).

**Figure 3:**
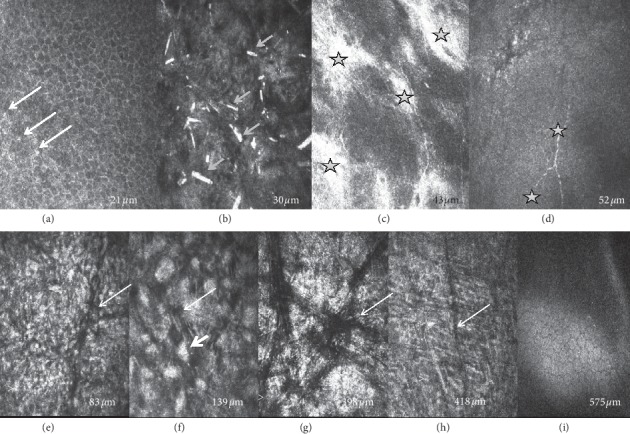
In vivo confocal microscopy images (400 × 400 *μ*m) at different scanning depths showing epithelial and stromal hyperreflective deposits and keratocyte damage in eyes with Schnyder dystrophy. (a) Epithelial wing cell layer with small round hyperreflective deposits (long thick arrows). (b) Basal epithelial cell layer with rod-shaped hyperreflective deposits (short arrows). (c) Both Bowman layer and the epithelial basement membrane (stars) appear to be undulated. (d) Thin subbasal nerves (stars). (e–h) stromal images show needle-shaped hyperreflective deposits (arrowheads), increased background reflectivity, stromal striae (thin arrows), absence of visible keratocyte nuclei, and rectangular deposits (thick arrow, only observed in patient #6). (i) Normal endothelium.

IVCM of the epithelium showed small round hyperreflective deposits with increased background reflectivity in five patients (Figure 3(a)), undulations of the epithelial basement membrane and Bowman layer, and thin subbasal nerves in four patients (Figures 3(c) and 3(d)). The subbasal nerve plexus was not detected in the remaining 3 patients. Epithelial cells featured normal shape in the superficial, intermediate, and basal cell layers. In one patient, epithelial deposits appeared to be rod-shaped (Figure 3(b)). The keratocyte nuclei were either rare or undetectable. Increased background reflectivity was observed in the anterior, mid, and posterior stroma. Most hyperreflective deposits appeared to be needle-shaped (Figures 3(e), 3(g), and 3(h)), and in one patient, rectangular-shaped deposits were noted as well (Figure 3(f)). Stromal striae were more easily visible with IVCM than with en face SD-OCT (Figures 3(e)–3(h)). Endothelial cells appeared normal (Figure 3(i)).

**Figure 4:**
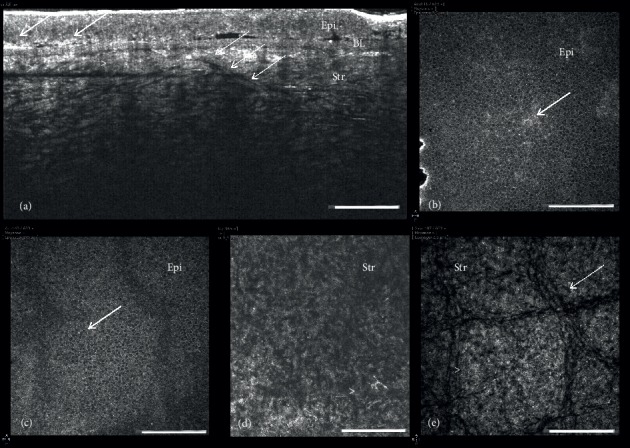
Full field-optical coherence tomography images at different scanning depths showing epithelial and stromal hyperreflective deposits and loss of keratocyte visibility in eyes with Schnyder dystrophy. (a) Cross-sectional reconstruction showing hyperreflective epithelial (arrows) and stromal (arrowheads) deposits and diffuse hyperreflectivity of the anterior stroma. (b-c) en face epithelial images showing epithelial deposit (arrows). Anterior (d) and posterior (e) stroma featuring stromal deposits (arrowheads) and stromal striae (thin arrow) with no visible keratocyte nuclei. Epi, epithelium; BL, Bowman layer; Str, stroma. Bars show 100 *μ*m.

Two corneal buttons were analyzed with FF-OCT after keratoplasty. Cross-sectional scans showed diffuse hyperreflective opacities in the anterior stroma (Figure 4(a)), and en face images revealed small round hyperreflective deposits in the epithelium (Figures 4(b)-4(c)) and small needle-shaped deposits in the anterior, mid, and posterior stroma (Figures 4(d)–4(e)). We also observed hyperreflective basement membrane. Keratocyte nuclei were undetectable, and stromal striae were also more easily visible compared with en face SD-OCT scans (Figure 4(e)).

**Figure 5:**
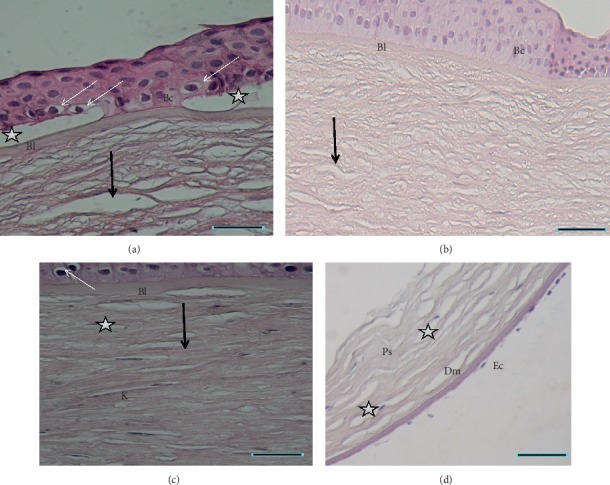
Corneal sections of Schnyder dystrophy stained with hematoxylin and eosin showing changes in the basal epithelial cell layer and stromal vacuolization and decreased keratocyte density. (a-b) the epithelium is formed by 4 to 7 layers. Columnar basal cells (Bc) feature apical nuclei surrounded by a clear halo and mild cytoplasmic vacuolization (thin arrows). Locally, the corneal epithelium can be detached (stars) from Bowman layer (Bl). Empty interlamellar vacuoles (arrows) are observed in the anterior and mid-stroma. The Bowman layer is preserved. (c) Mild cytoplasmic vacuolization (thin arrow) in the basal epithelial cells with preserved the Bowman layer. Some keratocyte nuclei K, as well as empty vacuoles (star), are noted in the anterior stroma. However keratocyte density is decreased from the anterior down to the posterior corneal stroma. (d) The posterior stroma (Ps) shows interlamellar vacuoles (stars). Descemet membrane (Dm) and endothelial cells appear to be normal. Scale bar show 20 *μ*m.

All patients underwent keratoplasty, and corneal buttons were processed for histology which showed cytoplasmic vacuolization of basal epithelial cells. Basal epithelial cell nuclei were surrounded by a clear halo (Figures 5(a)–5(c)) in five patients, and empty interlamellar stromal vacuoles were observed in all patients (Figure 5(a)). The Bowman layer was preserved (Figure 5(c)). Keratocyte density was low from the anterior down to the posterior corneal stroma. Endothelium and Descemet membrane appeared to be normal (Figure 5(d)).

**Figure 6:**
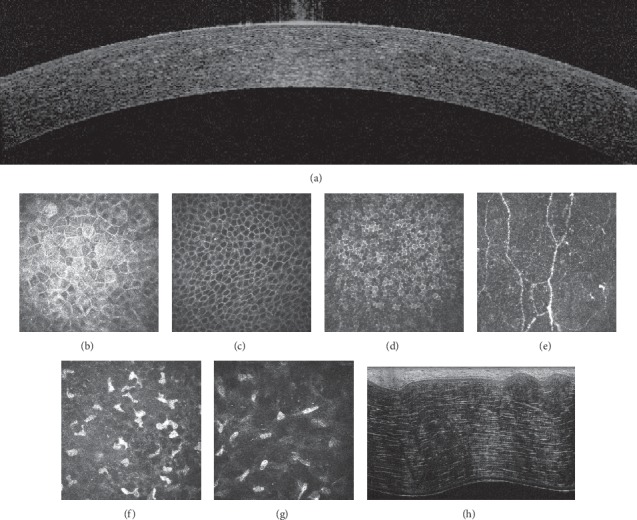
Spectral domain-optical coherence tomography (a), in vivo confocal microscopy (b–g), and ex vivo full-field optical coherence tomography of normal human corneas. A: cross-sectional SD-OCT scan. B: superficial epithelial cell layer. C: intermediate epithelial cell layer (wing cells). D: basal epithelial cell layer. E: subbasal nerve plexus. F: anterior stroma. G: posterior stroma. H: cross-sectional FF-OCT scan. No indicators of Schnyder dystrophy are visible.

Multimodal imaging of normal human corneas (Figure 6) did not reveal any of the indicators of Schnyder dystrophy revealed by SD-OCT, IVCCM, and FF-OCT.

The SD-OCT, IVCM, FF-OCT, and histology findings of all subjects are shown in Table 2.

Comparison with patient other eye did not reveal bilateral asymmetric disease presentation. Corneal central thickness was symmetric, and same SD-OCT and IVCM features of Schnyder dystrophy were observed in fellow eyes. Last, FF-OCT and histology were available only in the transplanted eyes.

## 4. Discussion

Corneal changes in SCD are age-dependent. Arcus lipoides tends to appear between 23 and 38 years of age, and midperipheral stromal haze develops in older patients [3]. Our patients featured late age of diagnosis and surgery. As our institution is a tertiary reference center, all our patients were referred at the time keratoplasty. As a matter of fact, the age of inclusion does not reflect the age of onset of the dystrophy. The youngest patient (52 years old) presented only with central disk-like corneal opacity with no arcus lipoides. Stromal haze was prominent in three of our oldest patients, aged 82, 84, and 92 years. According to Weiss et al., corneal crystals are found in approximately 50% of patients with SCD [7]. Only two of our seven patients presented with crystals. However, the number of patients examined was relatively low compared with that of the previous studies [13]. Absence of crystals renders the diagnosis of SCD more challenging and previous studies have shown the ability of modern imaging techniques such as SD-OCT and IVCM to identify the corneal features of SCD by allowing an in-depth evaluation of corneal structures [13–16].

Previously published data on SD-OCT in SCD included hyperreflective corneal opacities located under the epithelium and in the anterior stroma and a diffuse optical shadow from the stromal side [15]. This was consistent with our findings. We observed hyperreflective corneal opacities in the anterior stroma that extended to the limbus, except in our youngest patient who did not present with arcus lipoides (Figure 1(b)). In addition, we observed diffuse optical shadow from the stromal side in one patient who had prominent central corneal crystals on slit-lamp examination. Pachymetry was normal, and epithelial mapping revealed areas of irregular and thickened epithelium within the central 5 mm zone in all patients, which may be consistent with the presence of lipid and cholesterol deposits in the central epithelium as suggested by histology findings.

SD-OCT en face images showed diffuse high reflectivity in the epithelium, anterior, mid, and posterior stroma, which corresponded to the hyperreflective deposits observed with IVCM and FF-OCT at the same level. Presence of epithelial hyperreflectivity was consistent with presence of areas of thick and irregular epithelium on epithelial mapping. A previous study also reported highly reflective anterior corneal stroma on en face OCT scans suggesting crystal and/or lipid deposits in a SCD case, but the epithelium and Bowman layer seemed intact [17]. Stromal striae were visualized with SD-OCT en face scans in 3 patients. These striae correspond to undulations of the stromal lamellae associated with the viscoelastic behavior of the corneal stroma [18]. They are usually difficult to visualize with SD-OCT in normal corneas and easy to visualize with IVCM due to the higher resolution of the latter technique. Increased background reflectivity of the stroma in SCD may enhance the contrast between striae and the remaining stroma and makes the former easier to visualize with SD-OCT.

One patient presented an irregular epithelial basement membrane expanding into the epithelium which is a common finding in corneal epithelial basement membrane dystrophy [19]. Association of SCD with the latter dystrophy/degeneration may be the consequence of the high prevalence of the latter corneal disorder.

Previously published IVCM findings in SCD included damaged subepithelial nerves, increased background stromal reflectivity, and needle-shaped or rectangular deposits in the anterior stroma [14–16]. We observed small round hyperreflective deposits in the epithelium in four patients, as well as undulated basement membrane, but epithelial cells appeared normal in all layers. Evans et al. also observed round hyperreflective deposits in the epithelium and anterior stroma in an 8-year old child with SCD, as well as needle-shaped crystals, but posterior stroma was unaffected [16]. Vesaluoma et al. suggested that in advanced stages of SCD, large crystals are deposited in the anterior stroma and subbasal nerves are damaged, thus leading to decreased corneal sensation [20]. This observation was consistent with our findings. All seven patients, aged 52 years and older, presented with advanced forms of SCD, and although corneal sensation was not tested, IVCM scans revealed thin subbasal nerves in four patients, whereas subbasal nerves were undetectable in the remaining two patients assessed with IVCM.

Weiss showed that, although crystals were always subepithelial, SCD affected the entire thickness of the corneal stroma in the majority of patients [7]. This was corroborated by our findings. However, to the best of our knowledge, hyperreflective deposits in the posterior stroma have not yet been described with IVCM in SCD. Thanks to its high lateral resolution, IVCM showed hyperreflective deposits in the epithelium, needle-shaped crystals in the anterior stroma, sometimes coexisting with rectangular crystals, as well as hyperreflective material and increased background reflectivity in the mid and posterior stroma. Endothelium and Descemet membrane appeared normal in all IVCM images.

This study reports for the first time full-field optical coherence tomography findings in SCD. FF-OCT provides a cellular resolution similar to histology with no need for staining and fixatives [12]. It showed the hyperreflective deposits observed with IVCM in the epithelium and throughout the stroma. However, the needle-shaped and rectangular crystals were better highlighted with IVCM.

Documented histological findings in crystalline and noncrystalline forms of SCD included epithelial, stromal, and endothelial changes, characterized by the presence of vacuoles in basal cells, stroma, and endothelial cells [15, 21]. Interestingly, we observed similar cytoplasmic vacuolization in addition to nuclei surrounded by a clear halo in basal epithelial cells in five patients. The hyperreflective deposits in the epithelium evidenced with IVCM, en face OCT scans, and FF-OCT may correspond to lipid and/or cholesterol accumulation in the epithelium. This would be consistent with the clear halo surrounding nuclei and mild cytoplasmic vacuolization observed in the basal epithelial cells with histology. In fact, the histology preparation process including paraffin embedding dissolves lipids which appear as empty spaces in stained samples observed with light microscopy. Staining with oil red O or Sudan black would reveal lipids but it was not available in our series. Abnormal lipid and cholesterol accumulation in epithelial cells has also been documented with electron microscopy of corneas with SCD [9, 21, 22]. Unlike Nowinska et al., we did not observe any vacuoles in endothelial cells [15].

Our findings show that not only SCD results in stromal and epithelial lipid deposits but also it involves corneal cells as keratocytes, subbasal nerves, and basal epithelial cells were found to be affected by the disease process.

The main limitation of this study, besides the small number of patients included, was the lack of genetic testing for mutations in the UBIAD1 gene. However, SCD was confirmed by conventional histology following keratoplasty which provided diagnosis evidence. Another limitation of the study was that all the patients enrolled were old and none of subjects were in early disease or developing disease. Further longitudinal studies are needed to determine whether the multimodal imaging features of Schnyder dystrophy can be detected in the early stage of the disease.

## 5. Conclusion

High-resolution multimodal imaging techniques demonstrate the characteristic features of SCD which involve both the corneal epithelium and stroma and provide diagnosis confirmation even in eyes with no visible corneal crystals at slit-lamp examination.

## Figures and Tables

**Table 1:** Clinical data, slit-lamp finding, and pachymetry of seven eyes of seven patients with Schnyder corneal dystrophy.

Patient and eye	Age (y)	Gender	Treatment	BCVA	Corneal central thickness (*μ*m)	Slit-lamp findings	Epithelial mapping	
							Aspect	Max. thickness in the central 5-mm zone (*μ*m)
1 LE	75	F	DALK	20/50	582	Central stromal opacity and arcus lipoides	Irregular thickening	63
2 LE	84	M	PK	20/50	507	Central stromal opacity and arcus lipoides	Irregular thickening	62
3 RE	92	F	PK	20/2000	515	Central stromal opacity, stromal haze, and arcus lipoides	Irregular thickening	76
4 LE	52	M	DALK	20/50	569	Central disciform opacity, no arcus lipoides	Irregular thickening	63
5 LE	84	F	PK	20/100	586	Central stromal opacity, stromal haze, and arcus lipoides	Irregular thickening	62
6 LE	82	F	PK	20/100	550	Central opacity, crystals, stromal haze, and arcus lipoides	Irregular thickening	67
7 RE	63	F	PK	20/50	635	Central opacity, crystals, and arcus lipoides	Irregular thickening	67

LE = left eye, RE = right eye, F = female, M = male, BCVA = best-corrected visual acuity, DALK = deep anterior lamellar keratoplasty, PK = penetrating keratoplasty.

**Table 2:** Spectral domain optical coherence tomography (SD-OCT), in vivo confocal microscopy (IVCM), full-field optical coherence tomography (FF-OCT), and histology findings of seven eyes of seven patients with Schnyder corneal dystrophy.

Patient and eye	SD-OCT		IVCM		FF-OCT	Histology
	Cross-sections	En face sections	Epithelium	Stroma		
1 LE	Hyperreflective stromal opacities maximum in the central anterior stroma	Hyperreflectivity in epithelium and stroma; stromal striae visible in the posterior stroma	HD, BM undulations, thin subepithelial nerves, and increased background reflectivity	Undetectable keratocyte nuclei, HD, rare needle-shaped deposits, visible stromal striae	HD in epithelium and stroma, hyperreflective BM, undetectable keratocyte nuclei, visible stromal striae	Epithelial BC nuclei surrounded by a clear halo and mild cytoplasmic vacuolization, epithelium detached from BL, empty interlamellar stromal vacuoles
2 LE	Hyperreflective stromal opacities maximum in the central anterior stroma	Hyperreflectivity in epithelium and stroma; stromal striae visible in the posterior stroma	HD, BM undulations, thin subepithelial nerves and increased background reflectivity	Undetectable keratocyte nuclei, HD, rare needle-shaped deposits, visible stromal striae	HD in epithelium and stroma, hyperreflective BM, undetectable keratocyte nuclei, visible stromal striae	Normal epithelial BC, preserved BL, numerous interlamellar vacuoles, some keratocyte nuclei still visible
3 RE	Hyperreflective stromal opacities maximum in the central anterior stroma and in midperiphery	NA	HD, BM undulations and hyperreflectivity, thin subepithelial nerves	Undetectable keratocyte nuclei, HD, rare needle-shaped deposits, visible stromal striae	NA	Epithelium absent, preserved BL, numerous interlamellar vacuoles, some keratocyte nuclei still visible
4 LE	Hyperreflective stromal opacities maximum in the central anterior stroma	Hyperreflectivity in epithelium and stroma; stromal striae not visible	Normal epithelium, BM undulations, thin subepithelial nerves	Rare keratocyte nuclei, HD/high background reflectivity, rare needle-shaped deposits, visible stromal striae	NA	Epithelial BC nuclei surrounded by a clear halo and mild cytoplasmic vacuolization, preserved BL, empty interlamellar stromal vacuoles
5 LE	Hyperreflective stromal opacities maximum in the central anterior stroma and in mid-periphery; thickened epithelial basement membrane	Hyperreflectivity in epithelium and stroma; irregular basement membrane	HD, increased background reflectivity	Undetectable keratocyte nuclei, HD, rare needle-shaped deposits, visible stromal striae	NA	Epithelial parakeratosis, peripheral hyperplasia, preserved BL, anterior stroma fibrosis, stromal vacuoles
6 LE	Hyperreflective stromal opacities maximum in the central anterior stroma and in midperiphery	NA	HD, some needle-shaped deposits	Undetectable keratocyte nuclei, HD, needle-shaped and rectangular deposits, visible stromal striae	NA	Epithelial BC nuclei surrounded by a clear halo, preserved BL, empty interlamellar stromal vacuoles
7 RE	Hyperreflective stromal opacities maximum in the central anterior stroma in and midperiphery; posterior optical shadow	Hyperreflectivity in epithelium and stroma; stromal striae visible in the posterior stroma	Normal epithelium	Multiple tangled needle-shaped deposits in the anterior stroma, decreased keratocyte density	NA	BC nuclei surrounded by a clear halo and mild cytoplasmic vacuolization, preserved BL, empty interlamellar stromal vacuoles

LE = left eye, RE = right eye, HD = hyperreflective deposits, BM = basement membrane, BC = basal cells, BL = Bowman layer, NA = not available.

## Data Availability

The anonymized data used to support the findings of this study are available from the corresponding author upon request.
